# Epileptic Encephalopathies in Children

**DOI:** 10.1155/2013/505314

**Published:** 2013-08-22

**Authors:** Brahim Tabarki, Giangennaro Coppola, Elaine Wirrell

**Affiliations:** ^1^Division of Neurology, Department of Pediatrics, Prince Sultan Military Medical City, P.O. Box 7897, Riyadh 11159, Saudi Arabia; ^2^Clinic of Child and Adolescent Neuropsychiatry, Medical School, University of Salerno, S.Giovanni e Ruggi Hospital, Largo d'Ippocrate, 84100 Salerno, Italy; ^3^Divisions of Child and Adolescent Neurology, Department of Neurology, Mayo Clinic, 200 1st Street SW No. W4, Rochester, MN 55905, USA

Epileptic encephalopathies in children are severe disorders in which cognitive, sensory, and motor development is impaired by recurrent clinical seizures or prominent interictal epileptiform discharges. They include Ohtahara syndrome, early myoclonic epileptic encephalopathy, West syndrome, malignant migrating partial seizures in infancy, Dravet syndrome, Lennox-Gastaut syndrome, myoclonic atonic epilepsy, continuous spike wave in slow sleep, Rasmussen encephalitis, and other diseases. These epilepsies share several important characteristics: diverse causes; severe and frequent seizures; diffusely abnormal background activity on electroencephalograms that is often profound; medical intractability; and severe consequences for a normal development. Recently, several gene mutations have been found in several epileptic encephalopathies.

1. P. Jain and colleagues provide a summarizing article on the clinical evaluation and management of commonly encountered epileptic encephalopathies in children.
2. Wong-Kisiel and Nickels focus on electroencephalogram findings of childhood epileptic encephalopathy syndromes and provide sample illustrations.
3. J. Y. Yu and P. L. Pearl provide an overview of inborn metabolic errors associated with persistent brain disturbances due to highly active clinical or electrographic ictal activity. They also highlight the clues when to suspect a metabolic disease in a patient with epilepsy.
4. I. S. Fernández and colleagues review the chapter entitled Continuous Spikes and Waves during Sleep and provide detailed review about the electroclinical presentation and the management.
5. H. R. Kayyali et al. review the reported literature on the surgical approach to some of these epileptic encephalopathies in children.

These manuscripts represent an exciting and insightful snapshot of current knowledge of epileptic encephalopathies in children. State-of-the-art, existing challenges and emerging future topics are highlighted in this special issue. 

